# Age-Dependent Association among *Helicobacter pylori* Infection, Serum Pepsinogen Levels and Immune Response of Children to Live Oral Cholera Vaccine CVD 103-HgR

**DOI:** 10.1371/journal.pone.0083999

**Published:** 2014-01-15

**Authors:** Khitam Muhsen, Rosanna Lagos, Mardi K. Reymann, David Y. Graham, Marcela F. Pasetti, Myron M. Levine

**Affiliations:** 1 Center for Vaccine Development, Department of Medicine, University of Maryland School of Medicine, Baltimore, Maryland, United States of America; 2 Center for Vaccine Development, Department of Pediatrics, University of Maryland School of Medicine, Baltimore, Maryland, United States of America; 3 Centro Para Vacunas en Desarrollo, Hospital de Niños Roberto del Rio, Santiago, Chile; 4 Baylor College of Medicine, Michael E. DeBakey VA Medical Center, Houston, Texas, United States of America; Saint Louis University, United States of America

## Abstract

**Background:**

Through its effects on gastric secretion, we hypothesized that *Helicobacter pylori* infection may influence oral immunization. Accordingly, we examined the association between *H. pylori* infection, serum pepsinogen (PG) (measures for *H. pylori* gastritis) and vibriocidal antibody (a correlate of protection) seroconversion following oral immunization with CVD 103-HgR live cholera vaccine among children of different ages.

**Methods:**

Sera from 422 Chilean children who were vaccinated with a single dose of CVD 103-HgR were tested by ELISA for serum IgG antibodies to *H. pylori*, PG I and PG II levels and antibodies to *Shigella flexneri* 2a lipopolysaccharide and hepatitis A virus (as markers of low socioeconomic status and exposure to enteric pathogens).

**Results:**

The likelihood of vibriocidal antibody seroconversion following vaccination with CVD 103-HgR was significantly decreased in *H. pylori*-seropositive children age 6 months to 4 years with PG II>8 µg/L (adjusted OR 0.14 (95% CI 0.03–0.61; P = 0.009), and also in *H. pylori* seropositives with lower PG II level (adjusted OR 0.34, 95% CI 0.14–0.83; P = 0.017), compared to *H. pylori*-seronegatives. *H. pylori*-seropositive children aged 5–9 years with serum PG I>30 µg/L (indicating more severe gastritis) had higher odds of vibriocidal seroconversion than those with lower PG I levels (adjusted OR 4.41, 95%CI 1.26–15.38; P = 0.02). There was no significant association between exposures to *S. flexneri* 2a or hepatitis A virus and vibriocidal seroconversion.

**Conclusions:**

As *H. pylori* gastritis progresses with increasing pediatric age in developing country venues, changes in gastric secretion ensue that we believe explain the observed differences in age-related immune responses to immunization with live oral cholera vaccine. The effect of *H. pylori* and changes of gastric acid secretion on the immunogenicity of various oral vaccines should be studied in different developing, transitional and industrialized country settings.

## Introduction

Oral administration of vaccines constitutes a practical, simple, and safe method of immunization. With the exception of two non-living cholera vaccines (Dukoral® and Shanchol®), all other modern licensed oral vaccines have been live. These include attenuated poliovirus (trivalent, bivalent and monovalent formulations), three rotavirus vaccines (Rotashield™ [1], Rotarix™, and RotaTeq® [2]), *Salmonella* Typhi strain Ty21a [3] and attenuated *Vibrio cholerae* O1 strain CVD 103-HgR [4]–[6]. Despite their practical advantages, most of these vaccines have exhibited lower immunogenicity and efficacy when given to persons in developing countries compared to industrialized countries [2], [7].

The phenomenon of lower immunogenicity of CVD 103-HgR oral cholera vaccine in developing country populations has been intensively studied [4], [5], [7]–[13]. Whereas a single 5×10^8^ colony forming unit (CFU) dose of CVD 103-HgR elicited high titers of serum vibriocidal antibody (an immunologic correlate of protection) in 85–97% of US and European adults [4]–[6] and conferred significant protection against cholera [5], a one-log higher dose (5×10^9^ CFU) had to be administered to subjects in developing countries to achieve high vibriocidal antibody seroconversion rates [7], [9]–[12]. The correlates of diminished vibriocidal antibody response to CVD 103-HgR in developing country subjects [7] include an elevated serum vibriocidal antibody titer at baseline [9], proximal small bowel bacterial overgrowth (SBBO) [8] and low socioeconomic level [11]. Enhanced vibriocidal antibody responses (manifested as higher geometric mean titer [GMT]) were observed in subjects of O blood group [12], [13]. Interestingly, vibriocidal antibody responses could be elevated in non-O blood group subjects if they were treated with anti-helminthics prior to vaccination [13]. Despite these helpful insights, the full panoply of factors that affect the immune response to oral vaccines in developing country populations and their interplay is still not completely elucidated.

*Helicobacter pylori* a gram negative bacterium that colonizes the gastric mucosa, is acquired early in life in developing countries in association with low socioeconomic level and reaches a prevalence of >50% by 5 years of age [14]. *H. pylori* induces gastritis that mostly remains asymptomatic but that can alter gastric acid secretion, an important non-specific host defense against bacterial enteropathogens. Pepsinogen (PG) I and II, proenzymes for pepsin, are secreted into the gastric lumen by chief cells in the fundus and corpus of the stomach; PG II is also secreted by cells of the gastric antrum, as well as by Brunner's glands in the proximal duodenum. Approximately 1% of PG I and II enters the vascular system and can be detected in serum. Consequently, levels of serum PG I or PG II, or both, are increased in children with *H. pylori* gastritis [15]–[20], while the ratio of PG I∶PG II decreases as gastric inflammation progresses in severity [15]–[18]. In children and adults, serum pepsinogen levels and their ratio correlate well with the severity of gastric inflammation [15], [19], [20]. Importantly, even if no clinical symptoms are manifest, with increasing age progressive histological changes and gastric pathology develop [21]. Indeed, progressive damage of the gastric mucosa was observed in a 2-year follow-up of children with asymptomatic *H. pylori* gastritis [22].

We hypothesized that gastric colonization by *H. pylori* inducing gastric inflammation and possible changes in gastric acidity might impact the serological response to CVD 103-HgR through facilitating or inhibiting the passage of the vaccine strain through the stomach, to the duodenum, the attachment site *V. cholerae* O1. Therefore, we examined the association among evidence of *H. pylori* infection (the presence of IgG antibodies to *H. pylori* and to the CagA virulence protein encoded by a gene located in a chromosomal pathogenicity island), serum PG I and PG II levels (measures of gastric inflammation) and vibriocidal antibody seroconversion, following oral immunization with CVD 103-HgR in young children <5 years and in children 5–9 years of age. Since *H. pylori* may be a marker for other enteric infections that exhibit enhanced transmission in crowded, low socioeconomic level settings, we also examined whether past infection with hepatitis A and *S. flexneri* 2a (known to be prevalent in Santiago in the early 1990s [23]) correlate with the propensity to respond to CVD 103-HgR.

## Materials and Methods

### Vaccine

Attenuated *Vibrio cholerae* O1 strain CVD 103-HgR was licensed in the 1990s by many national regulatory agencies as a single-dose live oral cholera vaccine and was commercialized in two formulations, one containing ∼10^8^ CFU (Orochol® and Mutacol®) for travelers from industrialized countries and the other containing ∼10^9^ CFU (Orochol E®) for immunizing persons in developing countries. CVD 103-HgR is currently being re-commercialized by a new manufacturer (PaxVax, San Diego, CA).

### Study design and populations

We tested coded (anonymized) stored serum samples collected from children who were orally vaccinated with a single 5×10^9^ CFU dose of CVD 103-HgR in the course of 4 clinical trials carried out by the Centro para Vacunas en Desarrollo, Chile (CVD-Chile) in Santiago, Chile [8], [12], [24], [25] in the 1990s. Three trials assessed the immunogenicity of CVD 103-HgR in successively younger children of age 5–9 years [12], 2 to 4 years [24] and, finally, infants and toddlers 3 to 17 months [25]; we excluded sera of subjects <6 months of age from the infant study because of the inability to distinguish IgG *H. pylori* antibodies of maternal origin that might still be present. Another trial studied children aged 5 to 9 years who had fasting lactulose breath H_2_ tests to determine whether the presence of SBBO influenced the vibriocidal response to a dose of CVD 103-HgR [8]. Participants of the original trials were healthy children; children under antibiotic treatment were not enrolled.

Serum samples from 422 vaccinated pediatric subjects (47.3% female; 184<5 years and 238 5–9 years of age) were available for testing. The baseline specimen obtained prior to vaccination was tested for *H. pylori* antibodies (except for the SBBO trial from which we used the ∼day-10 post-vaccination samples since baseline specimens were no longer available from a proportion of the subjects).

### Laboratory methods

Serum vibriocidal antibody titers were measured upon completion of the clinical trials in the 1990s [8], [12], [24], [25]. Vibriocidal antibody seroconversion, defined as a >4-fold increase in serum vibriocidal antibody titer between baseline and 8–14 days after vaccination with one dose of CVD 103-HgR vaccine, was the outcome variable. In the current study serum IgG antibodies to *H. pylori* were measured using the Enzygnost® Anti-*Helicobacter pylori* II/IgG Enzyme Linked Immunosorbent Assay (ELISA) kit (Siemens Diagnostics Product GmbH, Marburg, Germany). Optical density (OD) values ≥0.250 were considered positive. The sensitivity of the kit in children is 92.7% and its specificity is 95.7%, and in those less than 6 years of age the respective values were 91.6% and 97.1% [26]. *H. pylori*-positive sera were thereupon tested for IgG antibodies to CagA using a commercial kit (Genesis Diagnostics, Cambridgeshire, UK). Samples were tested for *H. pylori* antibodies in a blinded manner without knowing their vibriocidal titers. The concentrations of serum PG I and II were measured using ELISA kits (Biohit, Helsinki, Finland) according to the manufacturer's instructions and PG I∶PG II ratios were calculated. Cut-offs of >8 µg/L for PG II [18] and <5 for the ratio of PG I∶PG II were used to indicate more severe degrees of gastric inflammation. A serum PG I level <25 µg/L denotes gastric atrophy, which is very rare in children. We utilized values of PG I>30 µg/L to indicate normal to high levels. To strengthen the classification of *H. pylori* serostatus in children less than five years of age, we followed the “2-tests strategy” [27] and classified children into 3 categories: i) *H. pylori* seronegatives; ii) positive for *H. pylori* IgG antibodies and having a serum PG II level ≤8 µg/L, or; iii) positive for *H. pylori* IgG antibodies and having a serum PG II level >8 µg/L, indicative of *H. pylori* gastritis. Sera were also tested by ELISA kit (Abnova Inc. Taipei, Taiwan) for hepatitis A antibodies following the manufacturer instructions, and for IgG antibodies to *S. flexneri* 2a lipopolysaccharide (LPS) [28]; titers for *S. flexneri* 2a were calculated from linear regression curves of serially diluted serum samples and expressed as ELISA units/mL [28]. The cutoff used to define positivity to *S. flexneri* IgG antibody was determined as 208.9 units/mL; it was calculated as the mean titer found among children ages 6–11 months (an age group of low incidence of shigellosis) plus 3 standard deviations (SD). The presence of *S. flexneri* and hepatitis A antibodies was considered an additional proxy for low SES and suboptimal hygiene standards [29] where the transmission of enteric pathogens is common.

Additional variables included age, sex and pre-vaccination (baseline) vibriocidal antibody titer (reflecting prior natural exposure to *V. cholerae* O1 or cross reacting antigens). ABO blood typing was performed in two of the studies [12], [24].

### Statistical analysis

Differences in the percentage of vaccinees with vibriocidal seroconversion after vaccination with CVD 103-HgR in *H. pylori* seropositive versus seronegative subjects, according to CagA IgG seropositivity, serum PG levels, age, sex, and presence of antibodies to hepatitis A and *S. flexneri* were examined using chi square test. Adjusted odds ratio (OR) and 95% Confidence Intervals (CIs) were obtained from logistic regression models. The variables entered in the multivariable analysis were baseline vibriocidal titers, age, sex, *H. pylori* seropositivity, PG I, PG II, PG I∶PG II ratio and presence of hepatitis A and *S. flexneri* antibodies. Analyses were stratified by age group (<5 years, 5–9 years), since the vibriocidal seroconversion, the prevalence of *H. pylori* infection and severity of gastritis may differ with age. One-way analysis of variance (ANOVA) and Bonferroni test were used to examine differences in the mean PG levels among *H. pylori* seropositive and seronegative children by age group with multiple comparisons correction. Correlations among the independent variables were assessed using Spearman coefficient. P<0.05 was considered statistically significant. Data were analyzed using SPSS version 20.

### Ethics

Since we used archived anonymized samples that were collected more than 15 years ago, without access to personal identifying data, the IRB of University of Maryland School of Medicine determined that the current study was exempt from full committee review and did not require that an updated consent be obtained from the individuals who provided the serum specimens.

## Results

### Overall vibriocidal antibody seroconversion

The pooled results of the four safety/immunogenicity studies of CVD 103-HgR in Chilean pediatric subjects that had been previously reported separately documented seroconversion of vibriocidal antibody following vaccination with CVD 103-HgR in 290 (68.7%) of the 422 children overall; however, seroconversion was somewhat higher in subjects aged 5–9 years (73.1%) than in children <5 years of age (63.0%) (P = 0.027). Children who failed to mount vibriocidal seroconversion had significantly higher baseline titers of vibriocidal antibody GMT = 18.3, SD±4.6) than those who did seroconvert (GMT = 13.9, SD±2.0) (P = 0.019). The GMT of vibriocidal antibody after vaccination was higher in persons of blood group O (GMT = 285.6, SD±18.0) than in non-O subjects (GMT = 144.8, SD±7.1) (P = 0.014), but the seroconversion rate was similar between the groups (70.1% vs. 64.4% P = 0.34). The percentage of vibriocidal seroconversion among males (71.4%) and females (66.4%) was similar (P = 0.27).

### *H. pylori* seropositivity, serum PG levels and vibriocidal seroconversion by age group

**Figure 1:**
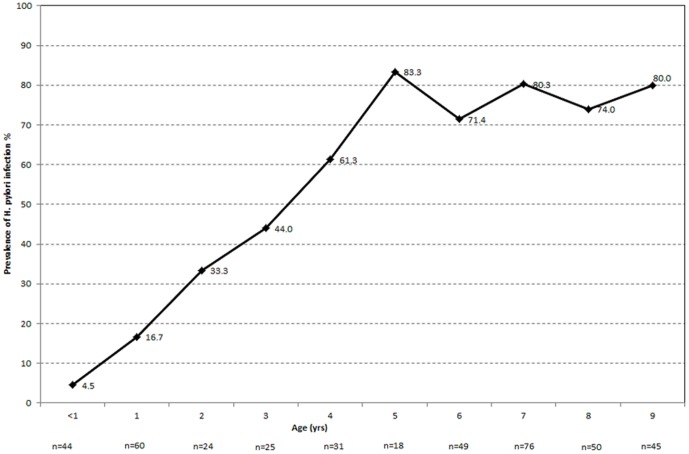
The Prevalence (%) of *Helicobacter pylori* infection by age.

In total, 234 of the 422 children (55.5%) were seropositive for *H. pylori* IgG antibodies. *H. pylori* seropositivity increased steeply during the first 5 years of life, from 4.5% to 61.3%, and stabilized at ∼70%–83% in children age 5–9 years (P<0.001) (figure 1).

Serum PG levels were measured to assess the degree of gastric inflammation (Table 1). In children <5 years of age, the mean PG II level was significantly higher among *H. pylori* seropositive than seronegative children. Among children aged 5–9 years, the PG I level was significantly increased in *H. pylori*-positive versus seronegative children. Moreover, the mean PG I level in *H. pylori*-seropositive children aged 5–9 years was significantly higher than in *H. pylori* positive children <5 years of age (Table 1).

**Table 1:** Mean serum pepsinogens levels in relation to the presence of IgG *H. pylori* antibodies and by age group.

	PG I	PG II	PG I∶PG II ratio
**Age<5 years**			
***H. pylori*** **-seronegative (n = 101)**	65.6 (58.4–72.8)	5.7 (4.4–7.0)	22.2 (18.3–26.0)
***H. pylori*** **-seropositive (n = 44)**	79.6 (68.2–91.0)	12.7 (6.9–18.4)*	19.8 (14.3–25.4)
**Age 5 to 9 years**			
***H. pylori*** **-seronegative (n = 42)**	69.3 (53.7–85.0)	9.2 (6.4–12.0)	12.7 (9.3–16.2)
***H. pylori*** **-seropositive (n = 162)**	103.4 (95.8–111.0)†	11.0 (9.6–12.3)	15.3 (13.0–17.6)

Data presented are mean levels and 95% CI. By ANOVA there was a significant difference between the age groups and according to serostatus (P<0.001 for PG I and PG II, and P = 0.002 for PG I∶PG II ratio).

Using the Bonferroni test that corrects for multiple comparisons a significant difference (P = 0.001) was noted in the serum PG II levels of *H. pylori*-seropositive vs. seronegative children aged <5 years.

Using the Bonferroni test, a significant difference (P<0.001) was also found for serum PG I levels in *H. pylori*-seropositive vs. seronegative children aged 5–9 years. Also, the mean serum PG I level among *H. pylori*-seropositive children aged 5–9 years was significantly higher than among *H. pylori*-seropositive children aged <5 years (P = 0.011). Other differences between the groups were not statistically significant.

An age-stratified analysis revealed that among children <5 years of age the rate of vibriocidal antibody seroconversion was markedly lower in *H. pylori*-infected (overall 46%, CagA-negative 46.4% and CagA-positive 45.5%) than in uninfected (69.4%) subjects (P = 0.005) (Table 2). In this age group *H. pylori* seropositive children with PG II≤8.0 µg/L had slightly higher (52%) vibriocidal serconversion than those with PG II>8.0 µg/L (47%). In contrast, among children aged 5–9 years, 77.3% of whom were *H. pylori*-positive, the vibriocidal seroconversion rate was similar whether they were *H. pylori* seropositive (72.3%) or seronegative (75.9%) (P = 0.59). However, among 5–9 year old children with a serum PG I level >30 µg/L, there was a clear trend towards a higher rate of vibriocidal seroconversion (P = 0.09) (Table 2). In this age group 83.5% of the subjects with a PG I level >30 µg/L were *H. pylori*-seropositive compared to only 45.5% of those with lower PG I levels (P<0.001).

**Table 2:** The association between *H. pylori* seropositivity, pepsinogen levels and vibriocidal seroconversion following vaccination with CVD 103-HgR.

	Total	Vibriocidal antibody seroconversion, n (%)	P value
**Children <5 years of age**			
**Serum IgG antibodies to ** ***H. pylori*** ** and CagA**			
*H. pylori*-negative	134	93 (69.4)	
*H. pylori*-positive (total)	50	23 (46.0)	
*H. pylori*-positive, CagA-negative	28	13 (46.4)	
*H. pylori*-positive, CagA-positive	22	10 (45.5)	0.005§
**Serum PG I levels** *			
≤30 µg/L	18	11 (61.1)	
>30 µg/L	127	84 (66.1)	0.67
**Serum PG II levels** *			
PG II≤8 µg/L	106	67 (63.2)	
PG II>8 µg/L	39	28 (71.8)	0.33
**PG I∶PG II ratio** *			
≤5	16	10 (62.5)	
>5	129	85 (65.9)	0.78
*H. pylori* positive and PG II>8 µg/L	15	7 (46.7)	
*H. pylori* positive and PG II≤8 µg/L	29	15 (51.7)	
*H. pylori* negative	101	73 (72.3)	0.012¶
**Hepatitis A antibodies** †			
Negative	108	72 (66.7)	
Positive	55	31 (56.4)	0.19
***S. flexneri*** ** IgG antibodies** ‡			
<209 ELISA units/mL	105	69 (65.7)	
≥209 ELISA units/mL	62	37 (59.7)	0.43
**Children aged 5–9 years**			
**Serum IgG antibodies to ** ***H. pylori*** ** and CagA**			
*H. pylori*-negative	54	41 (75.9)	
*H. pylori*-positive	184	133 (72.3)	
*H. pylori*-positive, CagA-negative	134	98 (73.1)	
*H. pylori*-positive, CagA-positive	50	35 (70.0)	0.59§
**Serum PG I levels** *			
≤30 µg/L	22	13 (59.1)	
>30 µg/L	182	138 (75.8)	0.09
**Serum PG II levels** *			
PG II≤8 µg/L	94	66 (70.2)	
PG II>8 µg/L	110	85 (77.3)	0.25
**PG I∶PG II ratio** *			
≤5	17	12 (70.6)	
>5	187	139 (74.3)	0.73
**Hepatitis A antibodies** †			
Negative	70	51 (72.9)	
Positive	130	97 (74.6)	0.78
***S. flexneri*** ** IgG antibodies** ‡			
<209 ELISA units/mL	35	28 (80.0)	
≥209 ELISA units/mL	174	129 (74.1)	0.46

P for the difference between *H. pylori*-seropositive versus seronegative children.

P for trend.

PG analysis is based on 145 and 204 that belonged to children aged <5 years and 5–9 years, respectively.

163 and 200 samples were available for hepatitis A testing, and.

167 and 209 samples were available for testing *S. flexneri* IgG antibody for children aged <5 years and 5–9 years, respectively.

The above associations were confirmed in multivariable analyses (Table 3). The odds of seroconversion were lowest in children <5 years of age who were seropositive for *H. pylori* and had PG II>8 µg/L (P = 0.009) (Table 3). Among older children 5–9 years of age, those with serum PG I levels >30 µg/L had 4-fold higher odds of seroconversion than subjects of this age with lower PG I levels (P = 0.02). This model also showed that each one log increase in baseline vibriocidal titer was associated with ∼35% lower likelihood of vibriocidal seroconversion following immunization with CVD 103-HgR. There was a significant positive, albeit weak, correlation between baseline titer of vibriocidal antibody and the presence of antibodies to *H. pylori* (r = 0.21, P<0.001), *S. flexneri* (r = 0.22, P<0.001) and hepatitis A (r = 0.17, P = 0.001). Whereas hepatitis A and *S. flexneri* seropositivity was significantly correlated with *H. pylori* infection (r = 0.22 and r = 0.51 respectively, P<0.001), the presence of these antibodies was not associated with vibriocidal seroconversion.

**Table 3:** Logistic regression models of the association between *H. pylori* infection, serum pepsinogen levels and vibriocidal seroconversion after immunization with a single oral dose of CVD 103-HgR.

	Unadjusted OR (95% CI)	Partially-adjusted OR (95% CI)†	Fully-adjusted OR (95% CI)	Pv
**Children <5 years of age (analysis 1)** *				
*H. pylori*-positive (vs. *H. pylori*-negative)	0.38 (0.18–0.79)	0.38 (0.18–0.81)	0.28 (0.12–0.64)	0.002
**Children <5 years of age (analysis 2)** **				
***H. pylori*** ** positive and PG II≤8** µg/L (vs. *H. pylori* negative)	0.41 (0.17–.96)	0.40 (0.17–0.93)	0.34 (0.14–0.83)	0.017
***H. pylori*** ** positive and PG II>8** µg/L (vs. *H. pylori* negative)	0.33 (0.11–1.01)	0.38 (0.12–1.14)	0.14 (0.03–0.61)	0.009
**Children aged 5–9 years** *				
Serum PG I>30 µg/L (vs. lower level)	2.17 (0.87–5.42)	2.23 (0.87–5.66)	4.41 (1.26–15.38)	0.02
Baseline vibriocidal titers (log scale)	-	-	0.65 (0.47–0.89)	0.007

Partially adjusted analysis, in addition to *H. pylori* serostatus/PG levels, age and sex were added to the analysis.

The following variables were entered: *H. pylori* infection, age (in years as a continuous variable), sex, PG I, PG II, PG I∶PG II ratio, baseline vibriocidal antibody titers (transformed into natural logarithm) *S. flexneri* 2a IgG and hepatitis A antibodies (as markers for environmental fecal contamination and low socioeconomic status). The final model of children aged <5 years included PG II and PG I∶PG II ratio but they were not significant, and gender (OR 0.42 95% CI 0.19–0.92, for Males vs. females). The final model of children aged 5–9 years included *S. flexneri* antibodies but it was not significantly associated with vibriocidal seroconversion.

In addition to *H. pylori*/PG II status, the following variables were entered to the analysis: age (in years as a continuous variable), gender, *S. flexneri* 2a IgG, hepatitis A antibodies and baseline vibriocidal antibody titers. The final model included the variables gender and hepatitis A, but they were not significantly associated with vibriocidal antibody seroconversion,

## Discussion

We have found evidence that *H. pylori* infection modulates the immune response to oral immunization with live oral cholera vaccine in a complex and age-related manner. In our Chilean pediatric cohorts *H. pylori* infection was acquired intensively during the first years of life, reaching a prevalence of 83.3% by 5 years of age (Figure 1), thereby corroborating earlier reports from Chile [30] and from various developing countries [14], [31]. Among young children with *H. pylori* infection of relatively short duration, gastritis is mild and presumably localized in the antrum and gastric acid secretion may often be increased [32]–[35]. This can explain why *H. pylori*-seropositive Chilean children <5 years of age who had a high serum PG II level manifested a 86% lower likelihood of vibriocidal antibody seroconversion following vaccination with CVD 103-HgR (P = 0.009), while *H. pylori* seropositive children with PG II<8 µg/L were ∼65% less likely to develop vibriocidal antibody seroconversion compared to young children lacking *H. pylori* antibodies (P = 0.017).

In the older Chilean 5–9 year olds, serum PG levels indicate that *H. pylori* infection was more chronic and extensive and had progressed to greater degrees of gastric inflammation that might involve the corpus, which in turn can be accompanied by hypochlorhydria [15], a well-recognized risk factor for the development of severe cholera [36]–[39]. Since long standing *H. pylori* gastritis can diminish gastric acidity [40], it is not surprising that studies from Bangladesh and Peru have reported a significantly increased risk of cholera in *H. pylori*-infected persons compared to uninfected ones [41]–[43]. Similarly, we observed that older (age 5–9 years) Chilean subjects with *H. pylori* infection or with higher serum PG I and PG II levels (indicating more severe gastritis, most likely consequent to chronic *H. pylori* infection) had higher odds of vibriocidal seroconversion following ingestion of live cholera vaccine.

The age-dependent associations we observed make sense when one takes into account the duration of *H. pylori* infection, the physiological consequences of its progression and the exquisite acid sensitivity of both wild type and attenuated *V. cholerae* O1 [39]. A study from Chile that compared gastric histological findings and immunological profile of children <12 years of age versus adults infected with *H. pylori* provides data that supports our age-dependent findings and the proposed explanations [44]. Lower inflammation scores of the gastric mucosa were observed among the children than adults, even though the bacterial load and percent CagA-positivity were similar among *H. pylori*-infected subjects, irrespective of age [44].

Whereas a high rate of vibriocidal antibody seroconversion was observed among Chilean 5–9 year olds, particularly among subjects with low baseline titers, the post-vaccination titers achieved are lower than those recorded in vaccinated young adults from industrialized countries [4]–[6]. We propose two phenomena that jointly contribute to explain this. First, hypochlorhydria also allows passage through the stomach of increased numbers of fecal organisms to which persons living in low socioeconomic environments are repetitively exposed. This can lead to environmental enteropathy [7], [45], which has been well described in Latin America in school age children living in poor environments [46] and which can diminish the vibriocidal antibody response to CVD 103-HgR [8]. Second, *H. pylori*, which may also affect the proximal duodenum, is a highly immunomodulating infection. It is quite possible that the proximal duodenum, where *V. cholerae* attaches, is altered so that the mucosa manifests both an up-regulated innate immune response and a Th1 pro-inflammatory suppressive environment that collectively inhibit the attenuated *V. cholerae* O1 vaccine organisms [33], [35].

Results of a clinical trial of live oral typhoid vaccine strain CVD 908-*htrA* in North American adults similarly showed that the serological response was significantly stronger in persons with *H. pylori* infection and chronic gastritis (based on serum PG levels) [47]. This provides confirmatory evidence for the observations made in the current study with live oral cholera vaccine in older Chilean children.

*H. pylori* infection is acquired in early childhood in populations living in crowded, low socioeconomic conditions, along with exposure to other enteric pathogens. Some may argue that this phenomenon, rather than *H. pylori* gastritis, leads to non-specific priming and stimulation of the immune system that affects the immunogenicity of CVD 103-HgR. We used serum antibodies to hepatitis A and *S. flexneri* 2a as surrogates for enhanced exposure to enteropathogens [29] and low socioeconomic level and adjusted for their impact; notably, this did not modify the association between *H. pylori* infection and vibriocidal seroconversion.

Our study has limitations. Serum IgG antibodies were measured to detect *H. pylori* infection, which is not ideal in very young children. However, the ELISA we used to detect *H. pylori* IgG antibodies has high sensitivity and specificity in young children [26]. Moreover, *H. pylori* seropositivity correlated significantly with serum pepsinogen levels, thus supporting the validity of our results. Lastly, applying the stringent 2-test strategy to indicate *H. pylori* gastritis in young children, i.e., *H. pylori* IgG seropositivity plus high PG II level, showed similar results.

Strengths of our study include the utilization of identical established methods to measure vibriocidal and *H. pylori* IgG antibodies and other markers in serum specimens from the four trials, and the same laboratory staff performed the assays. We hope our findings will encourage others to study the effects of *H. pylori* infection and its physiological consequences on the immunogenicity of oral vaccines in children and adults in both developing and industrialized country settings.
